# A Comparison in Physical Fitness Attributes, Physical Activity Behaviors, Nutritional Habits, and Nutritional Knowledge Between Elite Male and Female Youth Basketball Players

**DOI:** 10.3389/fpsyg.2021.685203

**Published:** 2021-05-31

**Authors:** Silvia Sánchez-Díaz, Javier Yanci, Javier Raya-González, Aaron T. Scanlan, Daniel Castillo

**Affiliations:** ^1^Faculty of Health Sciences, Universidad Isabel I, Burgos, Spain; ^2^Society, Sports and Physical Exercise Research Group (GIKAFIT), Department of Physical Education and Sport, Faculty of Education and Sport, University of the Basque Country (UPV/EHU), Vitoria-Gasteiz, Spain; ^3^Human Exercise and Training Laboratory, School of Health, Medical and Applied Sciences, Central Queensland University, Rockhampton, QLD, Australia

**Keywords:** team sports, eating, diet, performance, health, adolescent, gender

## Abstract

**Background:** Limited evidence exists comprehensively assessing physical fitness attributes, physical activity behaviors, nutritional habits, and nutritional knowledge according to sex in basketball players during early adolescence. Insight of this nature could be used to optimize the training process and lifestyles in young basketball players.

**Objective:** To compare physical fitness attributes, physical activity levels, nutritional habits, and nutritional knowledge between elite male and female basketball players under 14 years of age (U-14).

**Methods:** Twenty-three U-14 basketball players (male, *n* = 13 and female, *n* = 10) from the same elite basketball academy (Spanish Asociación de Clubes de Baloncesto [ACB] League) participated in this study. Physical fitness attributes were assessed using a basketball-specific test battery (countermovement jump, drop jump, linear sprint, Lane Agility Drill, 505 change-of-direction, and repeated-change-of-direction tests), while physical activity levels (Physical Activity Questionnaire for Adolescents, PAQ-A), nutritional habits (Turconi questionnaire), and nutritional knowledge (Turconi questionnaire) were assessed using questionnaires.

**Results:** Male players exhibited better physical fitness in all tests (p <0.001 to 0.036, effect size = −0.44 to −0.76, intermediate to strong) compared to female players. Male players also performed more physical activity in their leisure time (*p* = 0.036) than females. No significant differences in nutritional habits and nutritional knowledge were evident between sexes (*p* > 0.05). Of note, a high proportion of players declared never or only sometimes eating fruit (males: 23%; females: 40%) and vegetables (males: 46%; females: 70%). In addition, relatively poor nutritional knowledge was evident in all players with the group correctly answering <50% of nutritional questions overall (4.57 ± 1.88 out of 11 points, 42%) and according to sex (males: 4.07 ± 2.10, 37%; females: 5.20 ± 1.40, 47%).

**Conclusion:** These findings emphasize the necessity to perform individualized prescription of training stimuli across sexes to optimize the physical preparedness and development of youth basketball players. Additionally, strategies such as nutrition-focused education interventions may be necessary in this population given the low consumption of fruits and vegetables, as well as the poor nutritional knowledge observed in players.

## Introduction

Basketball is a highly demanding team sport, requiring players to adequately develop various physical fitness attributes for successful on-court performance (Castillo et al., 2021). In this sense, quantifying physical fitness attributes is important in the training process to detect deficits in players and consequently prescribe appropriate training strategies (Mancha-Triguero et al., 2019). Quantifying physical fitness attributes is especially important in basketball players during early adolescence (i.e., 12–14 years of age) who undergo dramatic body changes, such as sudden increases in height and body mass as well as alterations in motor control (Faigenbaum et al., 2009). Nevertheless, only one study has compared physical fitness attributes in basketball players under 14 years of age (U-14) according to sex. Specifically, Mancha-Triguero et al. (2021) observed that U-14 male basketball players who participated in the Spanish national championship performed better in single (male: 32.6 ± 2.6 cm; female: 25.5 ± 4.0 cm) and repeated (male: 26.4 ± 5.8 cm; female: 20.7 ± 4.3 cm) jumping tests while their U-14 female counterparts had better repeated-sprint ability (male: 14.1 ± 1.1 s; female: 13.8 ± 0.6 s). Given the limited evidence base to draw from, future studies on this topic seem necessary to facilitate the training process in basketball players during early adolescence for optimal progression into and across elite basketball academies.

Despite the known effect of training strategies on improving physical fitness in young basketball players (Schelling and Torres-Ronda, 2016), the importance of “invisible” training to optimize short- and long-term sports performance has also been highlighted (Mujika et al., 2018). “Invisible” training involves the application of strategies other than physical training, such as the physical activity performed during leisure time and nutritional habits (Mujika et al., 2018). In this sense, the physical activity performed outside of regulated sports training and competition can develop healthy lifestyle habits in young basketball players (López Villalba et al., 2016). Considering physical activity data recorded in German adolescents (*n*: 3,505, age: 12.0 ± 3.3 years) according to sex, a greater proportion of males have been shown to engage in self-reported physical activity in different domains outside the context of sports clubs (leisure-time physical activity outside of sports clubs, extracurricular physical activity and outdoor play) than females (52.6 vs. 46.9%, *p* = 0.010) (Reimers et al., 2019). Similarly, Barja-Fernández et al. (2020) observed males undertake significantly more leisure-time physical activity (e.g., aerobic, cycling, handball) than females (3.01 ± 0.84 vs. 2.79 ± 0.75, *p* = 0.04) among young Spanish adolescents (*n*: 662, age: 12.4 ± 0.9 years). Despite these trends in physical activity behaviors reported in European adolescents, no data exist demonstrating variations in physical activity between sexes specifically in young basketball players. Therefore, future research comparing physical activity levels during leisure time between sexes in U-14 basketball players are necessary to better understand lifestyle behaviors and further individualize training plans in this population.

Another fundamental aspect comprising “invisible” training is nutrition, which is not only vital for adequate growth and development, but also contributes to optimal recovery, performance, and injury risk in young athletes (Jeukendrup and Cronin, 2010). In addition, suitable nutritional habits can also positively affect psychological aspects such as self-concept and self-efficacy in young athletes (Balsalobre et al., 2014; Zaccagni et al., 2020). Thus, understanding the nutritional habits of basketball players during early adolescence, as well as sex-based differences in these behaviors, seems essential for the application of specific and effective nutritional strategies in young male and female basketball players. In this sense, several factors may contribute to poor nutritional habits in young athletes, including a lack of nutritional knowledge. In fact, Trakman et al. (2016) identified nutritional knowledge as a key modifiable determinant of dietary behaviors. Consequently, it is important to also understand the nutritional knowledge, and any underlying sex-based differences in knowledge, to explain nutritional habits in young basketball players and best inform specific intervention strategies to optimize their nutritional habits (e.g., nutrition education programs). However, no studies have explored nutritional habits and nutritional knowledge according to sex in young basketball players.

Given the importance of developing suitable fitness attributes and adopting appropriate activity and nutritional habits outside of training and competition in youth sports, it is essential to examine these aspects and quantify differences between males and females in young basketball players to best develop sex-specific training and lifestyle interventions in this population. Consequently, to address the gaps on this topic in youth basketball, this study aimed to quantify and compare physical fitness attributes, physical activity levels, nutritional habits, and nutritional knowledge between elite U-14 male and female basketball players. Based on findings in previous studies examining U-14 basketball players (Mancha-Triguero et al., 2021) and students during early adolescence (Reimers et al., 2019), we hypothesized that male U-14 players would possess better physical fitness and perform more physical activity outside of regular training and competition than females, and that U-14 females would have better nutritional habits and knowledge than males.

## Methods

### Study Design

A cross-sectional, observational study design was followed. In a single testing session, players had anthropometric measurements taken and completed a basketball-specific fitness test battery including jumping tests [countermovement jump (CMJ) and drop jump (DJ)], linear sprint tests, Lane Agility Drill test, 505 change-of-direction (COD) test, and repeated change-of-direction (RCOD) sprint test on an indoor basketball court (15–18°C, 60–70% relative humidity), with 5 min of passive, standing recovery applied between tests. Prior to testing, all players performed a standardized warm-up consisting of running at a moderate intensity for 5 min, followed by 5 min of jumps performed at progressively increasing intensities, 5 min of dynamic stretching exercises, and 3 min of 20-m running bouts performed at increasing intensities. Passive, standing recoveries were administered between efforts during the different warm-up tasks. All tests were carried out 3 days following official competition in the afternoon between 16:00 h and 19:00 h. Players were advised not to perform any physical exercise in the 2 days prior to testing and were given advice to ensure adequate hydration and nutritional status upon arrival for testing. Physical testing was conducted in groups of 3–4 players to ensure consistent recovery times could be administered between tasks across players during testing. Also, before physical testing, players completed questionnaires regarding physical activity behaviors, nutritional habits, and nutritional knowledge on their own and not in the presence of peers. Players were familiarized with the study protocol during training sessions across the month before the start of the study, including all physical fitness tests and questionnaires. Completion of the physical fitness testing and questionnaires took place during the same session during the in-season phase (October) of the 2020–2021 competitive season.

### Participants

Twenty-three U-14 basketball players (male, *n* = 13, age: 13.5 ± 0.3 yr, height: 168.1 ± 6.7 cm, body mass: 56.6 ± 12.5 kg, body fat composition: 14.6 ± 5.2%, muscle composition: 37.4 ± 7.7%, training experience: 6.0 ± 3.1 yr; and female, *n* = 10, age: 12.7 ± 0.5 yr, height: 161.1 ± 4.7 cm, body mass: 52.1 ± 7.2 kg, body fat composition: 24.1 ± 5.5%, muscle composition: 33.9 ± 1.7%, training experience: 5.0 ± 2.1 yr) participated in this study. All players belonged to the same elite basketball academy (i.e., Spanish Asociación de Clubes de Baloncesto [ACB] League) being members of teams competing at the highest competitive level in Spain for the U-14 age category. Players were included in the study if they completed all fitness assessments and questionnaires and had not missed >4 weeks of participation continuously in training during the 2 months prior to testing. All players were not consuming any medications or ergogenic supplements that may have altered performance. All players were undertaking on-court team training consisting of games-based and conditioning drills three times per week with each session typically lasting 75–90 min, as well as participating in one official match per week when testing took place. All players and their legal guardians were informed of the procedures, potential risks, and benefits of participation in the study before giving written informed assent (players) and consent (guardians). The study was performed in accordance with the Declaration of Helsinki (World Medical Association Declaration of Helsinki, 2013) and approved by the Ethics Committee of the University (Code: FUi1-P007).

### Physical Fitness Tests

#### Jumping Tests

Players performed two trials each of the CMJ and DJ to assess lower-limb power (Marshall and Moran, 2013). Each jump trial was separated by 45 s of passive, standing recovery. During CMJ trials, players were instructed to perform a downward movement followed by a complete, explosive extension of the lower-limbs, maintaining their hands on their hips while jumping as high as possible (Heishman et al., 2020). For DJ trials, players were instructed to step from a wooden box (30 cm high) and immediately following ground contact, jump for maximal height as quickly as possible (Marshall and Moran, 2013). A photocell system (Optojump, Microgate™, Bolzano, Italy) was used to measure jump height (cm) for each CMJ and DJ trial with the highest jump used for subsequent analysis in each test. The between-trial intraclass correlation coefficients (ICCs) for jump height attained during both tests was 0.97 in the current sample of players.

#### Linear Sprint Test

Players completed two trials of 20-m linear sprints at maximal effort to assess linear speed. Each sprint trial was separated by 120 s of passive, standing rest. Four pairs of photoelectric cells (Microgate™ Polifemo, Bolzano, Italy) were used to record sprint split times at 5, 10, and 20 m. The starting position was placed 0.5 m behind the first timing gate to avoid inadvertent triggering of timing, with players commencing each sprint on their own volition. The fastest time (s) for each split (irrespective of the trial) was used for subsequent analysis. The between-trial ICCs were 0.70, 0.67, and 0.75 for 5, 10, and 20-m sprint times in the current sample of players.

#### Change-Of-Direction (COD) Speed Tests

The Lane Agility Drill test and the 505 COD test were used to assess COD speed in players. In the Lane Agility Drill test, players started at the top left corner of the key, 0.2 m behind the free-throw line to avoid inadvertent triggering of timing, with players commencing each sprint on their own volition. Players faced the baseline throughout the entirety of the test. Initially, players ran 5.8 m to the baseline from the starting point. Players then side-shuffled 4.9 m to the right across the baseline before running backward to the top right corner of the free-throw line. Players then side-shuffled 4.9 m to the left where they touched the floor with their foot at the corner of the key (starting point), and then immediately completed the same circuit in the opposite direction (Raya-González et al., 2021). In the 505 COD test, players ran 15 m linearly from the starting position and performed a 180° turn, self-selecting the preferred lower-limb to initiate the change in direction. After changing direction, players ran as quickly as possible for a further 5 m back toward the starting position (Castillo et al., 2021). A photocell timing gate (Microgate™ Polifemo, Bolzano, Italy) was positioned 10 m from the starting position to capture performance time (s) across the 5 m immediately prior to and the 5 m immediately following the change in direction. Players completed two trials of each test with 90 s of passive, standing rest applied between trials. The fastest trial was used for subsequent analysis in each test. The ICCs were 0.90 for the Lane Agility Drill test and 0.74 for the 505 COD test in the current sample of players.

#### Repeated Change-Of-Direction (RCOD) Sprint Test

A single trial of the RCOD sprint test was administered consisting of 5 × 30-m shuttle sprints (15 + 15 m) interspersed with 30 s of passive, standing recovery between each sprint. Players started 0.5 m before the first timing gate and sprinted for 15 m, before touching a line on the floor with their preferred foot and returning to the starting position as fast as possible (Castillo et al., 2021). A single pair of photoelectric cells (Microgate™ Polifemo, Bolzano, Italy) were placed at the start line to record performance time (s) during each shuttle. The sum of all shuttle sprint times (total performance time during the RCOD sprint test) was calculated and used for subsequent analysis.

### Questionnaires

The questionnaires were conducted following a typical week in which players maintained their normal daily routines involving attendance at school on 5 days, three on-court training sessions, and participation in an official match during the weekend.

#### Physical Activity Questionnaire for Adolescents (PAQ-A)

The PAQ-A was applied to assess the physical activity behaviors of players outside of regular basketball training and competition across the entire week (Monday to Sunday) prior to testing. The PAQ-A consists of nine questions, each using 5-point Likert scales. The first six questions in the questionnaire assess the physical activity carried out in the last 7 days during leisure time, during physical education classes, during specific times on school days (lunch, afternoon, and night), and during the weekend. The last two questions of the questionnaire assess the level of physical activity carried out during the week and how often physical activity occurred on each day of the week. The final score attained in the PAQ-A is the average of the scores obtained in the first eight questions, while the final question is used to identify whether any circumstances that prevented usual physical activity occurred in the week that was analyzed. The PAQ-A was designed for and validated (ICC = 0.71 for total score) in males (*n* = 46) and females (*n* = 36) aged 13–18 years (Martínez-Gómez et al., 2009).

#### Dietary Questionnaire

The dietary questionnaire was applied to identify the nutritional habits and nutritional knowledge of players (Turconi et al., 2003). The dietary questionnaire was originally composed of 10 sections; however only three sections (i.e., B, C, and H) were deemed relevant for this study and thus used to assess player nutritional habits and nutritional knowledge. Furthermore, each section was slightly modified with the addition of a “non-reported” option for each item in each section for players to select if they did not feel compelled, were not comfortable, or did not know the precise amounts when answering a question. The three sections from the dietary questionnaire used in this study included:

- *Section B* is focused on the consumption frequency of common foods and beverages, consisting of 28 questions. The questions must be answered using categorical variables based on the perceived frequency of consumption by each player (i.e., always, often, sometimes, never).
- *Section C* is focused on nutritional habits related to breakfast contents, number of meals consumed in a day, daily consumption of fruit and vegetables, and daily consumption of soft drinks and alcoholic beverages. This section contains 14 questions with categorical variables based on the perceived frequency of consumption for 10 questions (i.e., always, often, sometimes, never) and open-ended responses able to be given for four questions. The maximum total score able to be attained in this section is 54.
- *Section H* is focused on nutritional knowledge, consisting of 11 questions. Each question is focused on important nutritional aspects including the function of specific macronutrients and micronutrients as well as the relevance of nutrition. Four answers are available for players to select in each question with only one answer being correct. A point is awarded for each correct answer, with no points awarded for incorrect answers. The maximum total score able to be attained in this section is 11.

### Statistical Analysis

Data are presented as mean ± standard deviations (SD) for quantitative variables or frequencies and percentages for qualitative variables. Considering the non-normal distribution of data detected by the Shapiro-Wilk test, Mann Whitney tests were used to examine differences in each physical fitness attribute, questions 1, 8, and the total score in the PAQ-A, and total scores in sections C and H in the dietary questionnaire between male and female groups. Practical significance was assessed by calculating Cohen's *r* effect size (ES) (Cohen, 1988), which were interpreted in magnitude as: small, <0.1; trivial, 0.11–0.30; intermediate, 0.31–0.50; and strong, >0.50 (Hopkins et al., 2009). Also, chi squared goodness-of-fit tests were used to examine the distribution of players for each item in the PAQ-A (questions 2–7) as well as sections B, C (questions 1–14), and H (questions 1–11) in the dietary questionnaire. Data analysis was carried out using the Statistical Package for Social Science (SPSS Statistics for Windows, version 25.0, IBM Corp., Armonk, N.Y., USA), with statistical significance set at *p* < 0.05.

## Results

The mean ± SD physical fitness attributes in the entire sample, as well as separately for male and female U-14 basketball players are shown in Table 1. Male players demonstrated significantly better jump heights (CMJ: *p* < 0.001, strong; DJ: *p* < 0.001, strong), linear sprint times (*p* < 0.001, strong), COD speed (Lane Agility Drill: *p* < 0.001, strong; 505 COD test: *p* = 0.001, intermediate), and RCOD time (*p* = 0.04, intermediate) than female players.

**Table 1:** Comparisons in physical fitness attributes between elite, male and female under 14 basketball players.

**Variable**	**Entire sample (*n =* 23)**	**Male players (*n =* 13)**	**Female players (*n =* 10)**	**Mean difference (%)**	***p*-value**	**Effect size, magnitude**
**Jump tests**						
Countermovement jump height (cm)	24.7 ± 5.6	27.6 ± 4.9	20.7 ± 3.8	25.1	**<0.001**	0.62, strong
Drop jump height (cm)	26.1 ± 6.4	30.0 ± 4.8	20.5 ± 3.6	31.7	**<0.001**	0.75, strong
**Linear Sprint test**						
5-m sprint time (s)	1.12 ± 0.08	1.07 ± 0.04	1.19 ± 0.06	−10.8	**<0.001**	−0.76, strong
10-m sprint time (s)	2.00 ± 0.14	1.92 ± 0.09	2.12 ± 0.12	−10.1	**<0.001**	−0.68, strong
20-m sprint time (s)	3.46 ± 0.40	3.23 ± 0.34	3.79 ± 0.23	−17.1	**<0.001**	−0.70, strong
**Change-of-direction (COD) speed tests**						
Lane Agility Drill time (s)	15.31 ± 1.00	14.76 ± 0.58	16.10 ± 0.97	−9.1	**<0.001**	−0.66, strong
505 COD test time (s)	2.68 ± 0.20	2.60 ± 0.15	2.80 ± 0.22	−7.7	**0.001**	−0.48, intermediate
**Repeated change-of-direction (RCOD) sprint test**						
RCOD sprint test time (s)	34.13 ± 2.06	33.36 ± 1.56	35.23 ± 2.27	−5.6	**0.036**	−0.44, intermediate

*Bolded p-value indicates statistical significance at p < 0.05*.

Responses to the PAQ-A in the entire sample, as well as separately for male and female U-14 basketball players are presented in Table 2. While male players reported significantly higher physical activity during leisure time compared to female players (*p* = 0.036), no significant differences were evident between sexes in all other questionnaire items nor PAQ_total_.

**Table 2:** Comparisons in Physical Activity Questionnaire for Adolescents (PAQ-A) results between elite, male and female, under 14 basketball players.

**Question**	**Entire sample (*n =* 23)**					**Males (*n =* 13)**					**Females (*n =* 10)**					***p*-value**
PAQ1_mean_ Activities	0.48 ± 0.27					0.59 ± 0.31					0.34 ± 0.14					**0.04**
PAQ2 Physical education	Never	Hardly ever	Sometimes	Often	Always	Never	Hardly ever	Sometimes	Often	Always	Never	Hardly ever	Sometimes	Often	Always	
	0	0	8.7	56.5	34.8	0	0	0	61.5	38.5	0	0	20	50	30	0.24
PAQ3 Lunch	Sit	Walk	Play little	Play a lot	Play intensely	Sit	Walk	Play little	Play a lot	Play intensely	Sit	Walk	Play little	Play a lot	Play intensely	
	69.6	21.7	4.3	4.3	0	76.9	15.4	7.7	0	0	60	30	0	10	0	0.41
	None	Once a week	2–3 a week	4 a week	>4 a week	None	Once a week	2–3 a week	4 a week	>4 a week	None	Once a week	2–3 a week	4 a week	>4 a week	
PAQ4 4–6 pm	21.7	13	30.4	21.7	13	23.1	0	30.8	38.5	7.7	20	30	30	0	20	0.08
PAQ5 6–10 pm	13	13	47.8	17.4	8.7	7.7	7.7	61.5	15.4	7.7	20	20	30	20	10	0.63
PAQ6 Weekend	8.7	22.7	43.5	17.4	8.7	7.7	15.4	38.5	23.1	15.4	10	30	50	10	0	0.58
PAQ7 Week intensity	Little	1–2 a week	3–4 a week	5–6 a week	>6 a week	Little	1–2 a week	3–4 a week	5–6 a week	>6 a week	Little	1–2 a week	3–4 a week	5–6 a week	>6 a week	
	21.7	39.1	34.8	4.3	0	23.1	30.8	38.5	7.7	0	20	50	30	0	0	0.70
PAQ8_mean_ Diary frequency	1.80 ± 0.63					1.84 ± 0.69					1.74 ± 0.57					0.66
PAQ_total_	1.63 ± 0.52					1.73 ± 0.60					1.50 ± 0.40					0.32

*PAQ1_mean_ Activities, Frequency of physical activities during leisure time across the last 7 days; PAQ2 Physical education, Frequency of being physically active during physical education sessions at school across the last 7 days; PAQ3 Lunch, Type of physical activity before and after lunch across the last 7 days; PAQ4 4–6 pm, Frequency of being physically active immediately after school during the last 7 days; PAQ5 6–10 pm, Frequency of being physically active between 6 and 10 pm across the last 7 days; PAQ6 Weekend, Frequency of being physically active during the last weekend; PAQ7 Week intensity, Weekly frequency of performing physical activity in leisure time; PAQ8_mean_ Diary frequency, Frequency of daily physical activity for each day of the week; PAQ_total_, Total score obtained across the first eight questions in the questionnaire; Mann-Whitney U-tests were applied to questions 1, 8, and total score, which contain data presented as mean ± standard deviation, while Chi-squared tests were applied to all other questions which contain data presented as percentages. Bold value indicates significance at p < 0.05*.

Results from the dietary questionnaire regarding the nutritional habits and nutritional knowledge of the entire sample as well as separately for male and female U-14 basketball players are shown in Table 3 and Table 4, respectively. No significant differences (*p* > 0.05) were found between sexes in nutritional habits or nutritional knowledge. However, descriptive data showed a high proportion of players never or only sometimes eat fruit (males: 23%; females: 40%) and vegetables (males: 46%; females: 70%). Furthermore, players demonstrated relatively poor nutritional knowledge with <50% of questions being answered correctly across the entire sample (4.57 ± 1.88 out of 11 points, 42%) and in each sex (males: 4.07 ± 2.10, 37%; females: 5.20 ± 1.40, 47%).

**Table 3:** Comparisons in nutritional habits between elite, male, and female, U-14 basketball players.

**Question**	**Entire sample (*n =* 23)**					**Male players (*n =* 13)**					**Female players (*n =* 10)**					***p*-value**
C1 Breakfast	Never	Sometimes	Often	Always	NR	Never	Sometimes	Often	Always	NR	Never	Sometimes	Often	Always	NR	
	4.3	0	0	95.7	0	7.7	0	0	92.3	0	0	0	0	100	0	0.37
C2 Beverage breakfast	Tea	Juice	Chocolate	Milk	NR	Tea	Juice	Chocolate	Milk	NR	Tea	Juice	Chocolate	Milk	NR	
	0	0	0	95.7	4.3	0	0	0	92.3	7.7	0	0	0	100	0	0.37
C3 Eat breakfast	Cheese	Pizza	Bread	Fruit	NR	Cheese	Pizza	Bread	Fruit	NR	Cheese	Pizza	Bread	Fruit	NR	
	0	0	95.7	0	4.3	0	0	92.3	0	7.7	0	0	100	0	0	0.37
	Never	Sometimes	Often	Always	NR	Never	Sometimes	Often	Always	NR	Never	Sometimes	Often	Always	NR	
C4 Fruit	13	17.4	30.4	34.8	4.3	7.7	15.4	30.8	46.2	0	20	20	30	20	10	0.54
C5 Vegetables	8.7	47.8	26.1	13	4.3	15.4	30.8	23.1	23.1	7.7	0	70	30	0	0	0.16
C6 Cake	21.7	52.2	13	8.7	4.3	23.1	53.8	7.7	7.7	7.7	20	50	20	10	0	0.83
C7 Wine, beer	60.9	30.4	0	8.7	0	61.5	23.1	0	15.4	0	60	40	0	0	0	0.36
C8 Three meals	0	0	21.7	78.3	0	0	0	15.4	84.6	0	0	0	30	70	0	0.40
C9 Diet	Monotony	Different on weekend	Different sometimes	Different all days	NR	Monotony	Different on weekend	Different sometimes	Different all days	NR	Monotony	Different on weekend	Different sometimes	Different all days	NR	
	4.3	0	8.7	87.0	0	7.7	0	0	92.3	0	0	0	20	80	0	0.18
C10 Diet based on	Different all days	Carbohydrate	Lipids	Protein	NR	Different all days	Carbohydrate	Lipids	Protein	NR	Different all days	Carbohydrate	Lipids	Protein	NR	
	56.5	0	4.3	34.8	4.3	61.5	0	7.7	30.8	0	50	0	0	40	10	0.51
C11 Snacks	Sweets	Fried	Bread	Fruit	NR	Sweets	Fried	Bread	Fruit	NR	Sweets	Fried	Bread	Fruit	NR	
	8.7	21.7	26.1	30.4	13	7.7	23.1	30.8	15.4	23.1	10	20	20	50	0	0.30
C12 Beverages	Juice	Wine, beer	Refresh	Water	NR	Juice	Wine, beer	Refresh	Water	NR	Juice	Wine, beer	Refresh	Water	NR	
	0	0	8.7	91.3	0	0	0	15.4	84.6	0	0	0	0	100	0	0.19
	Never	Sometimes	Often	Always	NR	Never	Sometimes	Often	Always	NR	Never	Sometimes	Often	Always	NR	
C13 Milk	0	0	4.3	95.7	0	0	0	0	100	0	0	0	10	90	0	0.24
C14 Water	0	13	26.1	56.5	4.3	0	7.7	15.4	69.2	7.7	0	20	40	40	0	0.31
C Total	44.78 ± 3.70					45.39 ± 3.86					44.00 ± 3.53					0.45

*All questions were obtained from section C in the Turconi questionnaire; NR, not reported; Chi-squared tests were applied to questions 1–14, while a Mann-Whitney U test was applied to the total score*.

**Table 4:** Comparisons in nutritional knowledge between elite, male and female, U-14 basketball players.

**Question**	**Entire sample (*n =* 23)**		**Male players (*n =* 13)**		**Female players (*n =* 10)**		***p*-value**
	***Correct (%)***	***Incorrect (%)***	***Correct (%)***	***Incorrect (%)***	***Correct (%)***	***Incorrect (%)***	
H1 Carbohydrates	56.5	43.5	61.5	38.5	50.0	50.0	0.58
H2 Fiber	17.4	82.6	7.7	92.3	30.0	70.0	0.16
H3 Fat	8.7	91.3	7.7	92.3	10.0	90.0	0.85
H4 Protein	56.5	43.5	46.2	53.9	70.0	30.0	0.25
H5 Calories	56.5	43.5	61.5	38.5	50.0	50.0	0.58
H6 Energy	47.83	52.17	53.85	46.15	40.0	60.0	0.51
H7 Vitamins and minerals	4.3	95.7	0	100	10.0	90.0	0.24
H8 Balanced diet	21.74	78.26	76.9	23.1	80.0	20.0	0.86
H9 Daily energy expenditure	65.2	34.8	53.8	46.2	80.0	20.0	0.19
H10 Biological foods	56.5	43.5	46.2	53.8	70.0	30.0	0.25
H11 Transgenic foods	21.7	78.3	15.4	84.6	30.0	70.0	0.40
H Total	4.57 ± 1.88		4.07 ± 2.10		5.20 ± 1.40		0.24

*All questions were obtained from section H in the Turconi questionnaire; total score is presented as mean ± standard deviation for the number of correctly answered questions*.

## Discussion

A better understanding of the physical fitness attributes, physical activity behaviors, nutritional habits, and nutritional knowledge of elite male and female basketball players participating in a youth basketball academy is essential to optimize their health and development in prescribing specific and effective training and nutritional strategies. In this regard, elite U-14 male basketball players exhibited better physical fitness across all tests (i.e., a test battery consisting of jumping, linear sprint, and change-of-direction tests) and performed more physical activity in their leisure time compared to elite U-14 female basketball players. In contrast, no significant differences were observed between sexes in nutritional habits and nutritional knowledge; however, male and female players reported a low consumption of fruit and vegetables and demonstrated relatively poor nutritional knowledge across a range of nutritional concepts.

Regarding physical fitness, the results of this study showed that elite male U-14 basketball players had superior jump heights (i.e., CMJ and DJ), linear speed (i.e., 5, 10, and 20 m- sprint times), COD speed (i.e., Lane Agility Drill and 505 COD tests), and RCOD speed (i.e., RCODAtotal) than female players. These results coincide with those reported in U-14 Spanish national basketball players revealing that male players (*n* = 33) possess superior lower-body power (i.e., abalakov jump and multi-jump test) and repeated-sprint capacity (i.e., repeat sprint ability test) compared to U-14 female players (*n* = 12) (Mancha-Triguero et al., 2021). This finding is supported with previous literature since sex differences in athletic performances start to increase around the age associated with the onset of puberty in males (12–13 years) (Handelsman, 2017). Also, the superior physical fitness in U-14 male players across many of the fitness tests we examined may be attributed to them possessing greater absolute and relative fat-free mass than females in early adolescence as females have been shown to experience a decline in relative fat-free mass prior to puberty (McCarthy et al., 2014). Furthermore, physical fitness attributes (linear sprint time and RCOD ability) have been shown to be significantly associated (*p* < 0.05; *r* = −0.60 to −0.63) with external load variables (total distance, high-speed running distance, and number of jumps performed) during simulated matches in elite U-14 male basketball players (Castillo et al., 2021) and with performance index rating during matches in elite U-14 male (CMJ height, 20-m linear sprint time, and Agility *T*-test time, *r* = −0.25 to 0.23, *p* < 0.01) and female (CMJ power, *r* = 0.16, *p* < 0.05) basketball players (Ramos et al., 2019). Given this demonstrated importance of physical fitness attributes for in-match activity and performance, a thorough understanding of differences in physical fitness between male and female U-14 players may inform the development of training stimuli to optimize the physical preparedness of players across both sexes to meet the demands of competition.

While the players in the present study underwent a rigorous training routine participating in an elite basketball academy, an understanding of their physical activity level outside the sports context is also of interest given its positive effects on athletic performance (Mujika et al., 2018). In this way, identifying discrepancies in physical activity performed during leisure time between sexes during early adolescence is needed to understand specific trends in lifestyle behaviors during this phase of life and for the development of specific training strategies. In this study, no significant differences were reported in most items contained in the PAQ-A. Only higher PAQ1_mean_ activities, that is, how often players engaged in activities identified in the PAQ-A (skipping rope, cycling, jogging, racket sports, soccer, etc.) during their leisure time, were evident in male players compared to female players. Similar to these findings, higher participation in physical activity outside of sports clubs has been reported in males compared to females in German adolescents (*n* = 2,117, age: 12.0 ± 3.3 years, 52.6 vs. 46.9%, *p* = 0.01) (Reimers et al., 2019) and in Spanish adolescents (*n* = 662, age: 12.4 ± 0.9 years, 3.01 ± 0.84 AU vs. 2.79 ± 0.75 AU, *p* = 0.04) (Barja-Fernández et al., 2020). The greater leisure-time physical activity we observed in elite U-14 male basketball players compared to female players might be attributed to males experiencing greater encouragement from significant reference people (i.e., parental models) to engage in physical activity than females during early adolescence (Dixon et al., 2008). However, we observed no significant differences between sexes in physical activity behaviors during physical education classes, on school days, and during the weekend, suggesting participation in an elite basketball academy may lessen the differences in leisure-time physical activity between males and females compared to the wider population as shown in European adolescents (Reimers et al., 2019; Barja-Fernández et al., 2020). As such, it seems that basketball practice in an elite academy may have had a positive influence on equating the practice of physical activity in wider social contexts across sexes in young players.

In addition to adequate physical fitness, a balanced and appropriate diet is essential to ensure optimal growth and performance are attained in adolescent basketball players (Iglesias-Gutiérrez et al., 2005, 2012). This is the first study to analyze differences in nutritional habits according to sex in basketball players during early adolescence, with no significant differences in nutritional habits observed between males and females. However, closer examination of the nutritional habits of the players examined in this study reveal some notable findings. Specifically, 30% of the entire sample (23% of males and 40% of females) never or sometimes eat fruit, while 57% of the entire sample (46% of males and 70% of females) never or sometimes eat vegetables. As such, considering the global recommendation of consuming 5 fruits and vegetables daily to prevent chronic diseases such as overweight, obesity, diabetes, or cardiovascular diseases (World Health Organization, 2003), the limited consumption of fruit and vegetables we observed in elite U-14 basketball players is concerning from a health perspective. From a performance perspective, consumption of fruits and vegetables has been shown to influence body composition (fat mass and fruit intake, *r* = −0.21, *p* < 0.01; fat free mass and vegetable intake, *r* = 0.25, *p* < 0.01) and physical fitness (progressive aerobic cardiovascular endurance run performance and vegetable intake, *r* = 0.22, *p* < 0.01) in University students (21.5 ± 1.5 years) (López-Sánchez et al., 2019). Research has indicated that the consumption of fruits and vegetables can delay or prevent the appearance of chronic non-communicable diseases, that is, diseases associated with unhealthy lifestyle habits (e.g., obesity, type II diabetes) (Lampe, 1999; Tian et al., 2018). These benefits are mainly related to the nutritional composition of foods, including vitamins, minerals (essential nutrients), and dietary fiber. By incorporating fruits and vegetables into the daily diet in adolescents, the intake of fats, sugars, and salt are typically reduced, which can help prevent weight gain and reduce the risk of developing overweight or obesity later in adulthood (World Health Organization/Food and Agriculture Organization, 2003).

Like our findings regarding player nutritional habits, no significant differences in nutritional knowledge were found between sexes in elite U-14 basketball players. These insights are novel for youth basketball players and are necessary to identify knowledge deficits regarding nutritional concepts that can be addressed in targeted educational interventions tailored to both sexes (Bird and Rushton, 2020). While these data are the first on this topic in basketball players, contradictory findings have been reported in other athletic populations. For instance, Ali et al. (2015) observed greater nutritional knowledge (*p* < 0.05) in female (19.3 ± 0.7 years) compared to male athletes (21.0 ± 1.8 years) who were involved in football, volleyball, cross-country, basketball, swimming, and other sports activities than males (21.0 ± 1.8 years) using a nutrition knowledge and dietary habits questionnaire. In contrast, Manore et al. (2017) reported no significant differences in nutritional knowledge (*p* = 0.08; females = 45%, males = 56% correct answers) between sexes in high school soccer players under 15 years of age using a nutritional knowledge questionnaire. Irrespective of sex, relatively poor nutritional knowledge was demonstrated across the entire sample of elite U-14 basketball players we examined (42% correct responses). Likewise, some relevant nutrition concepts such as fiber, fat, vitamins, minerals, balanced diet, and transgenic foods were poorly understood across players. Taking into account that an adequate nutritional knowledge could promote better nutrional habits (Muderedzwa and Matsungo, 2020) and consequently better health and physical conditioning (Nikolaidis and Theodoropoulou, 2014), nutrition education interventions are likely needed in elite U-14 basketball players to enhance nutritional habits. Further research on this topic is encouraged to identify whether the poor nutritional knowledge we observed represents that of the wider elite adolescent basketball player population.

This study is not exempt from limitations, which should be acknowledged. First, given U-14 players from a single elite basketball academy were strictly recruited in this study, a relatively small sample size was used in each group (males and females). Accordingly, future studies should expand on this work examining larger samples of players and adolescent players from other age categories and levels of play (i.e., international). Secondly, endurance capacity was not measured in this study. Due to the documented importance of aerobic fitness in accomplishing high-intensity running distances during matches in elite, adolescent (18.2 ± 0.5 years) male basketball players (Ben Abdelkrim et al., 2010), further studies comparing fitness attributes between sexes in youth basketball players should include aerobic fitness testing protocols (e.g., Yo-Yo Intermittent Recovery Test, 30–15 Intermittent Fitness Test). Thirdly, data were acquired at a single timepoint in the season and does not capture changes in fitness, physical activity behaviors, or nutritional habits across the season. Consequently, future research should conduct fitness testing and questionnaire assessments at different timepoints throughout the season to understand temporal changes in fitness, physical activity behaviors, and nutritional habits to implement specific strategies at different phases across the competitive season. Fourthly, analysis of nutritional habits was limited to broad patterns of dietary behaviors, whereas further understanding of daily energy expenditure and macronutrient intake would allow for more detailed insight to develop comprehensive nutrition intervention strategies promoting healthy nutritional habits in adolescent basketball players. Finally, maturity status was not able to be measured in this study. Future research on this topic is encouraged to identify the maturity status of players where permissible to understand its role on the variables measured in this study.

## Conclusions

Elite U-14 male basketball players had greater physical fitness and underwent more physical activity during leisure time compared to female players, suggesting individualized prescription of training stimuli across sexes should be adopted to optimize the physical preparedness and development of players. Additionally, while nutritional habits and nutritional knowledge were similar between sexes, players exhibited low consumption of fruits and vegetables as well as relatively poor nutritional knowledge. Consequently, strategies such as education interventions may be necessary to improve the nutritional knowledge of elite basketball players in early adolescence.

## Data Availability Statement

The raw data supporting the conclusions of this article will be made available by the authors, without undue reservation.

## Ethics Statement

The studies involving human participants were reviewed and approved by Universidad Isabel I. Written informed consent to participate in this study was provided by the participants' legal guardian/next of kin.

## Author Contributions

SS-D led the project and developed and revised the original manuscript. DC and JR-G analyzed and interpreted the data and developed and revised the original manuscript. AS revised the original manuscript. JY developed the statistical report and revised the original manuscript. All authors contributed to the article and approved the submitted version.

## Conflict of Interest

The authors declare that the research was conducted in the absence of any commercial or financial relationships that could be construed as a potential conflict of interest.
